# The prognosis of clinical stage IIIa non‐small cell lung cancer in Taiwan

**DOI:** 10.1002/cam4.6357

**Published:** 2023-07-26

**Authors:** Ya‐Fu Cheng, Jing‐Yang Huang, Ching‐Hsiung Lin, Bing‐Yen Wang

**Affiliations:** ^1^ Division of Thoracic Surgery, Department of Surgery Changhua Christian Hospital Changhua Taiwan; ^2^ Institute of Medicine, Chung Shan Medical University Taichung Taiwan; ^3^ Center for Health Data Science Chung Shan Medical University Hospital Taichung Taiwan; ^4^ Department of Recreation and Holistic Wellness MingDao University Changhua Taiwan; ^5^ Department of Internal Medicine, Division of Chest Medicine Changhua Christian Hospital Changhua Taiwan; ^6^ Institute of Genomics and Bioinformatics National Chung Hsing University Taichung Taiwan; ^7^ Department of Post‐Baccalaureate Medicine College of Medicine, National Chung Hsing University Taichung Taiwan

**Keywords:** heterogenicity, non‐small cell lung cancer, outcomes, stage IIIa, Taiwan

## Abstract

Lung cancer is the leading cause of cancer death. The treatment of stage IIIa remained the most controversial of all stages of non‐small cell lung cancer (NSCLC). We reported on the heterogenicity and current treatment strategies of stage IIIa NSCLC in Taiwan. This study is a retrospective analysis using data from the Taiwan Society of Cancer Registry between January 2010 and December 2018. 4232 patients with stage IIIa NSCLC were included. Based on cell type, the best 5‐year OS (40.40%) occurred among adenocarcinoma victims. The heterogenicity of T1N2 had the best 5‐year OS (47.62%), followed by T4N0 (39.82%), and the others. Patients who underwent operations had better 5‐year OS (over 50%) than those who did not (less than 30%). Segmentectomy (75.28%) and lobectomy (54.06%) showed better 5‐year OS than other surgical methods (less than 50%). In multivariable analysis, young age, female, lower Charlson Comorbidity Index score, adenocarcinoma cell type, well differentiated, T1N2/T4N0 heterogenicity, treatment with operation, and segmentectomy/lobectomy/bilobectomy were significant factors. In conclusions, the heterogenicity of T1N2 had the best outcomes followed by T4N0. Patients received surgical treatment revealed much better outcomes than those did not. As always, multimodal therapies with individualized treatment tend to provide better survival outcomes.

## Graphical abstract

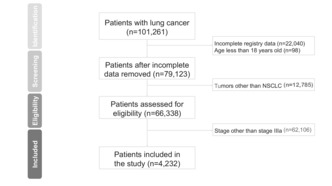

**Novelty & Impact Statements**

The treatment of stage IIIa remained the most controversial of all stages of non‐small cell lung cancer (NSCLC). The heterogenicity of stage IIIa NSCLC is seldom reported. In this cohort study using Taiwan Society of Cancer Registry data, 4232 patients were enrolled between 2010 and 2018. The heterogenicity of T1N2 had the best outcomes, followed by T4N0. Patients received surgical treatment revealed much better outcomes than those did not.

## 1 INTRODUCTION

Lung cancer is identified as the leading cause of cancer death and the second most diagnosed cancer in both genders worldwide.^1^ In 2020, there were over 2 million new lung cancer patients diagnosed and 1.8 million lung cancer deaths were reported. Around 84% of lung cancer cases are attributed to non–small cell lung cancer (NSCLC). In Taiwan, the incidence of lung cancer increased from 16.5 to 37 cases per 100,000 from 1986 to 2017. The age‐standardized mortality rate also slightly increased in these years.^2^ Over 70% of these lung cancer patients will die within 5 years. Early detection and prompt surgical treatment are vital to improve the mortality rate.

The treatment of stage IIIa remained the most controversial of all stages of NSCLC. About 5%–10% of NSCLC was diagnosed in stage IIIa.^3^, ^4^ Stage IIIa NSCLC is subdivided into the N0, N1, and N2 nodal stages with varying tumor sizes, classified as T1–T4.^5^ This very broad and diverse population might be treated with various methods and obtain different outcomes. In general, multimodal treatments are believed to provide a better 5‐year overall survival (OS) rate.^6^ There were many studies that compared the trimodality treatment (surgery, chemotherapy and radiotherapy), bimodality treatments and other combinations.^7^, ^8^, ^9^, ^10^ They resulted in different outcomes and suggestions due to variations in patient characteristics. The treatment recommendations for stage IIIa NSCLC even vary based on the patient's age.^11^ However, there was seldom studies focused on the heterogenicity of stage IIIa NSCLC. The treatment strategies, surgical methods, and outcomes were quite different between T1N2, T4N0, or other heterogenicity of stages. We assume that the clinical N0, N1, and N2 status in stage IIIa NSCLC patients need different treatments instead of the same therapeutic method. The clinical T4N0 status might need larger resection with lobectomy while neoadjuvant and adjuvant therapy take minor effect of outcomes. On the contrary, neoadjuvant and adjuvant therapy might take major effect of outcomes to the clinical T1N2.

In this study, we reported the current treatment strategies for stage IIIa NSCLC in Taiwan. The outcomes of different heterogenicity subgroups in stage IIIa, modalities of treatment and surgical methods were described. We aimed to provide more information about the effective treatment strategies for stage IIIa NSCLC.

## 2 MATERIALS AND METHODS

### 2.1 Patient population and selection

We conducted a retrospective cohort study and obtained data from the Taiwan Society of Cancer Registry (TSCR) over a 9‐year period, January 2010 to December 2018, in order to find out appropriate treatments for each group of patients. The TSCR data include Taiwan's entire population of 23 million people, and all cancer cases in Taiwan have been recorded in a uniform format since 1979. All the patients were confirmed by tissue diagnosis and provided a clinical stage of NSCLC. The study was approved by the Institutional Review Board in our institution (IRB‐221211), and informed consent from all participants was waived.

Patients who were diagnosed with lung cancer and had morphology codes ranging from 8000 to 9581 were included. A total of 101,261 patients with lung cancer were identified. The exclusion criteria were described as follows: incomplete registry data, age less than 18 years old when diagnosed, tumors other than NSCLC, clinical stage other than stage IIIa lung cancer.

Regarding the evaluation of the clinical stage, the National Health Insurance of Taiwan covered all preoperative staging workups, including chest computed tomography (CT) scans, upper abdomen positron emission tomography (PET)/CT scans with contrast, bronchoscopies, and brain magnetic resonance imaging (MRI). For the mediastinum lymph node staging, endobronchial ultrasound‐guided transbronchial needle aspiration (EBUS‐TBNA) was performed on the PET/CT positive patients and the patients who had lymph nodes larger than 1 cm.

The outcome measures for our study were 5‐year OS rate and median survival time. The OS was calculated as the time from tissue confirmation of malignancy to either death or December 2019.

### 2.2 Statistical analyses

Continuous variables are presented using mean ± SE. Survival curves were plotted by the Kaplan–Meier method, and between‐group differences in OS were assessed using the stratified log‐rank test. Univariate and multivariate analyses were performed with the Cox proportional hazards model. Hazard ratios and associated 95% confidence intervals were estimated. Covariates were selected based on clinical judgment. The following factors were included into analyses: age, gender, CCI score, cell type, tumor grade, TN stage, treatment, and operative method. All calculations were performed using IBM SPSS Statistics for Windows, Version 22.0 (IBM Corp.). Statistical analysis with a *p*‐value less than 0.05 was considered statistically significant.

## 3 RESULTS

In total, 4232 patients with stage IIIa NSCLC were included. The basic data on patient characteristics are shown in Table 1. There were 2082 patients (49.19%) over 70 years old when diagnosed and 1860 patients (43.95%) who were 50–69 years old. We found that patients over 70 years old had much worse 5‐year OS and median survival (18.26%; 16.72 months) than other groups. Male predominance was noted (*n* = 2972, 70.22%), and males had worse outcomes than females (5‐year OS: 25.74% vs. 43.75%).

**TABLE 1:** Patient demographic data and univariate survival analysis.

Variables		Patients	5‐Year survival rate, % (mean ± SE)	Median survival time, months (mean ± SE)	*p*‐Value
All		4232	31.10 ± 0.76	26.89 ± 0.72	–
Age					<0.0001
18–49		290	45.59 ± 3.10	44.88 ± 4.98	
50–69		1860	43.35 ± 1.23	44.32 ± 2.77	
≧70		2082	18.26 ± 0.91	16.72 ± 0.54	
Gender					<0.0001
Male		2972	25.74 ± 0.86	20.81 ± 0.68	
Female		1260	43.75 ± 1.51	47.10 ± 2.15	
CCI score					<0.0001
	≦2	2716	34.86 ± 0.98	30.99 ± 1.32	
	3–5	1340	24.80 ± 1.25	21.28 ± 1.02	
	>5	176	21.81 ± 3.37	16.50 ± 3.32	
Cell type					<0.0001
	AD	2175	40.40 ± 1.14	42.40 ± 1.69	
	SqCC	1698	20.25 ± 1.03	15.50 ± 0.59	
	LCC	211	20.27 ± 2.89	13.39 ± 1.56	
	Others	148	34.53 ± 4.38	36.76 ± 2.25	
Grade					<0.0001
	Well differentiated	203	62.46 ± 3.52	98.30 ± 7.52	
	Moderately differentiated	1183	38.29 ± 1.47	38.03 ± 1.92	
	Poorly differentiated	923	30.89 ± 1.57	25.02 ± 1.47	
	Undifferentiated	32	40.63 ± 8.68	22.00 ± 5.66	
	Unknown	1516	19.43 ± 1.06	17.45 ± 0.59	
TN stage					<0.0001
	T1N2	455	47.62 ± 2.53	54.71 ± 5.21	
	T2N2	1341	30.83 ± 1.36	28.48 ± 1.09	
	T3N1	455	31.21 ± 2.32	25.50 ± 2.14	
	T3N2	944	20.43 ± 1.36	15.85 ± 0.77	
	T4N0	713	39.82 ± 1.97	34.82 ± 2.13	
	T4N1	324	21.21 ± 0.26	19.24 ± 1.86	
Treatment					<0.0001
	None	403	4.88 ± 1.12	9.07 ± 0.71	
	OP alone	475	57.20 ± 2.41	78.21 ± 6.36	
	Neoadjuvant + OP + adjuvant	363	55.03 ± 2.76	74.57 ± 4.69	
	OP + adjuvant	1188	49.61 ± 1.59	59.40 ± 2.22	
	CRT	393	21.86 ± 2.28	19.92 ± 1.52	
	CT ± target therapy	714	8.88 ± 1.14	13.75 ± 0.72	
	RT alone	300	6.36 ± 1.50	9.89 ± 0.73	
	Target therapy	256	23.85 ± 2.96	27.83 ± 3.06	
	Others	140	8.11 ± 2.54	13.85 ± 1.37	
OP method					<0.0001
	None	2227	11.88 ± 0.74	13.91 ± 0.48	
	Wedge resection	257	46.05 ± 3.30	51.95 ± 9.16	
	Segmentectomy	56	75.28 ± 6.47		
	Lobectomy	1546	54.06 ± 1.38	68.85 ± 2.81	
	Bilobectomy	81	48.93 ± 5.86	59.02 ± 5.11	
	Pneumonectomy	65	36.88 ± 6.33	44.29 ± 6.91	

Abbreviations: AD, adenocarcinoma; CCI, charlson comorbidity Index; CRT, chemoradiotherapy; CT, chemotherapy; LCC, large cell carcinoma; OP, operation; RT, radiotherapy; SqCC, squamous cell carcinoma.

There were 2716 patients (64.17%), 1340 patients (31.66%), and 176 patients (4.15%) with CCI scores less than two, from three to five, and over five, respectively. The 5‐year OS declined with the elevation of CCI score (34.86%, 24.80%, and 21.81%). The cell type was adenocarcinoma (AD) predominant (*n* = 2175, 51.39%), followed by squamous cell carcinoma (SqCC) (*n* = 1698, 40.12%), large cell carcinoma (LCC) (*n* = 211, 4.98%) and others (*n* = 148, 3.49%). AD resulted in better 5‐year OS (40.40%) than SqCC (20.25%) and LCC (20.27%). The most common tumor grade was moderately differentiated (*n* = 1183, 27.95%). The tumor grade of well differentiated showed better 5‐year OS (62.46%) than moderately differentiated (38.29%), poorly differentiated (30.89%), and undifferentiated (40.63%).

The different TN stages of stage IIIa NSCLC also showed different outcomes. There were 455 patients (10.47%) with T1N2, 1341 (31.68%) with T2N2, 944 (21.83%) with T3N2, 455 (10.52%) with T3N1, 324 (7.49%) with T4N1 and 713 (16.49%) with T4N0. It can be seen that T1N2 corresponded with the best 5‐year OS (47.62%), followed by T4N0 (39.82%), T3N1 (31.21%), T2N2 (30.83%), T4N1 (21.21%), and T3N2 (20.43%). Although all the patients were classified as stage IIIa NSCLC, the T1N2 patients revealed a 5‐year OS rate that was more than twice the 5‐year OS rates of the T4N1 and T3N2 patients.

Turning to the treatment options, most patients received an operation with adjuvant therapy (*n* = 1188, 28.07%). It is interesting that the best 5‐year OS corresponded with operation alone (57.20%), followed by operation with both neoadjuvant and adjuvant therapy (55.03%), operation with adjuvant therapy (49.61%), target therapy (23.85%) and ch emoradiotherapy (CRT) (21.86%). It is easy to understand that operable stage IIIa NSCLC provided better survival than non‐operable tumors. However, our data pointed out that operation alone provided better 5‐year OS than bimodality and trimodality treatments. We suggested there might be a selection bias in that the operation alone group might include more T4N0 patients with better outcomes whereas bimodality and trimodality groups might include more T4N1 and T3N2 patients with worse outcomes.

Regarding operative methods, the most common operation type was lobectomy (*n* = 1546, 36.53%). It provided better 5‐year OS (54.06%) than wedge resection (46.05%), bilobectomy (48.93%) and pneumonectomy (36.88%). Our data indicated that segmentectomy provided significantly higher 5‐year OS (75.28%). However, there were only 56 patients that underwent segmentectomy.

**FIGURE 1:**
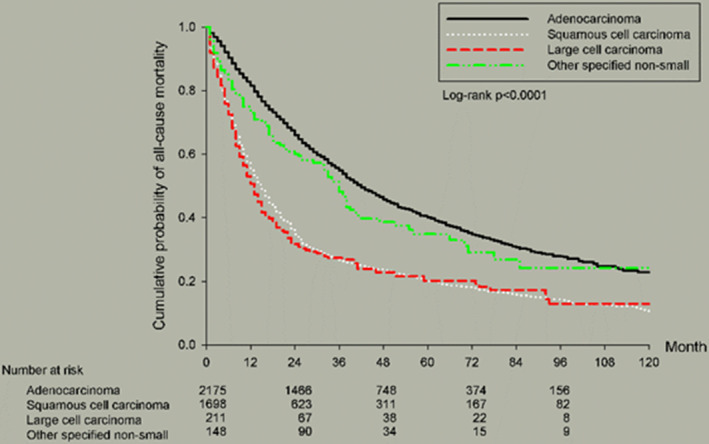
Kaplan–Meier survival curves by cell type for all patients with stage IIIa NSCLC.

**FIGURE 2:**
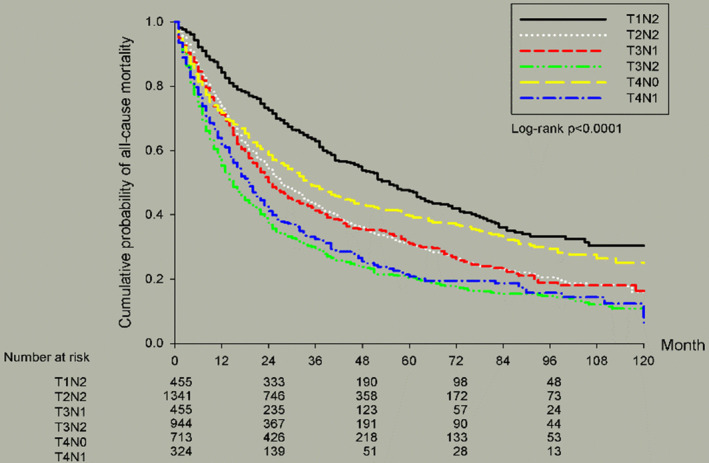
Kaplan–Meier survival curves by TNM status for all patients with stage IIIa NSCLC.

**FIGURE 3:**
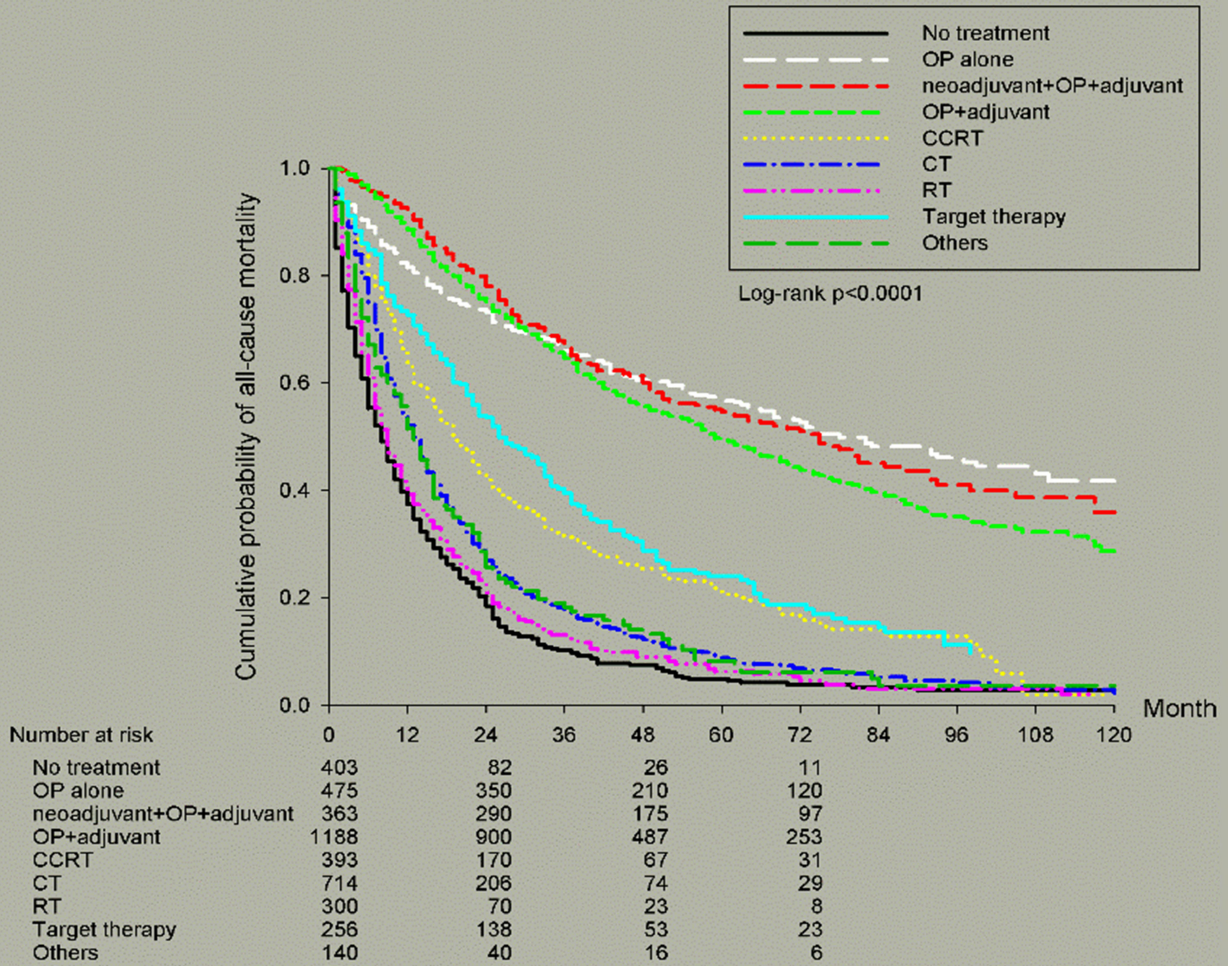
Kaplan–Meier survival curves by treatment option for all patients with stage IIIa NSCLC.

**FIGURE 4:**
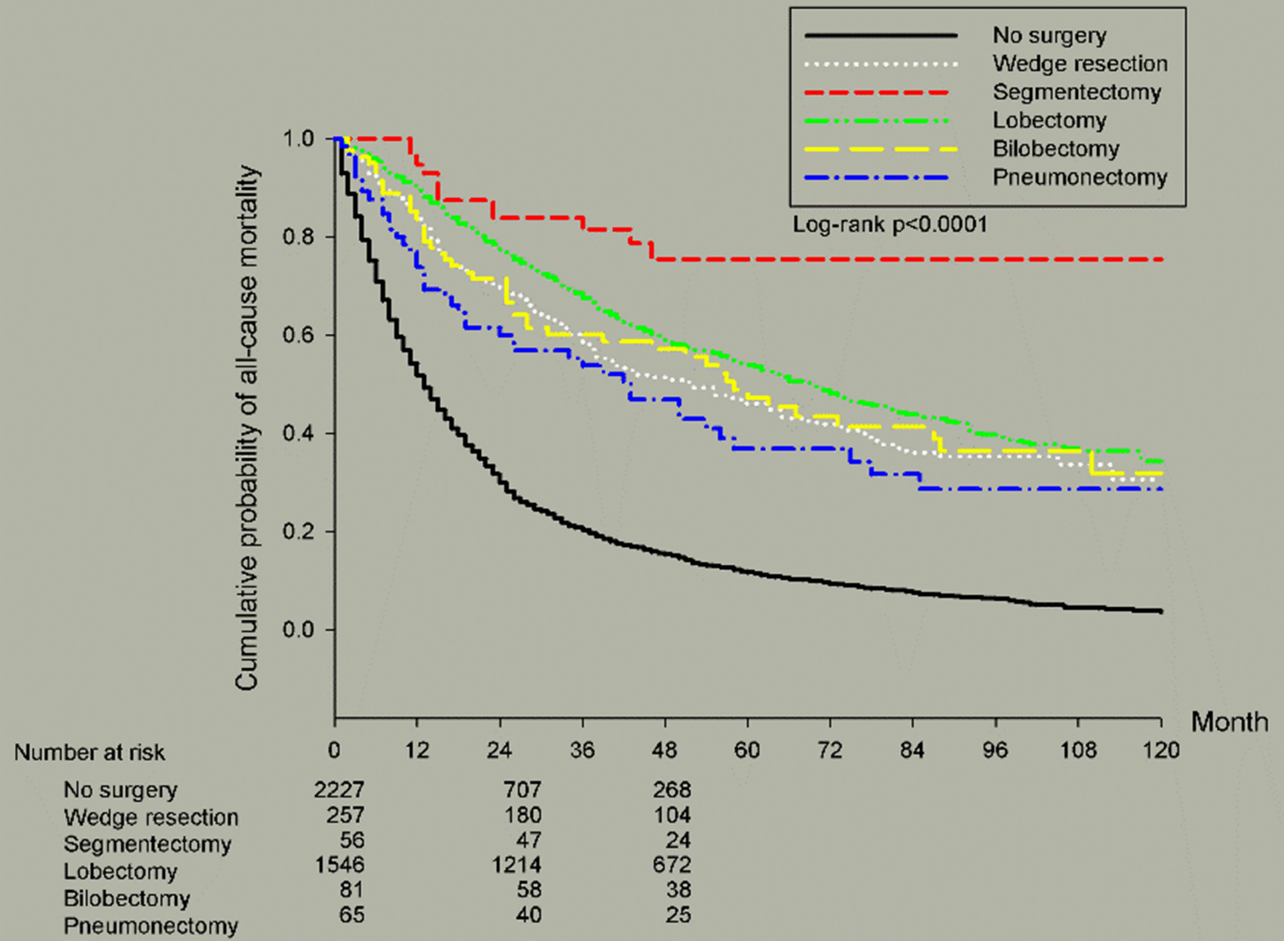
Kaplan–Meier survival curves by surgical method for all patients with stage IIIa NSCLC.

Figure 1 showed survival curves based on different cell types of stage IIIa NSCLC. AD resulted in better OS than SqCC and LCC (*p* < 0.0001). Figure 2 illustrated survival curves based on different TN statuses of stage IIIa NSCLC. As we mentioned previously, T1N2 and T4N0 resulted in better OS than other TN stages (*p* < 0.0001). Figure 3 presented survival curves based on different treatment options for stage IIIa NSCLC. Patients who underwent operations possessed better 5‐year OS than any other treatment without operation. Figure 4 depicted survival curves based on different surgical methods for stage IIIa NSCLC. Segmentectomy provided better OS than lobectomy, wedge resection, bilobectomy and pneumonectomy.

Both the univariable and multivariable linear regression models for OS were analyzed in Table 2. In univariable analysis, young age, female, lower CCI score, AD cell type, well differentiated, T1N2 stage, treatment with operation, and surgical method other than none were found to be statistically associated with better survival. In multivariable analysis, young age, female, lower CCI score, AD cell type, well differentiated, T1N2/T4N0 stage, treatment with operation, and segmentectomy/lobectomy/bilobectomy were significant factors.

**TABLE 2:** Univariate and Multivariate analyses of overall survival.

Variables	Univariate analysis			Multivariate analysis		
	HR	95% CI	*p*‐Value	HR	95% CI	*p*‐Value
Age						
18–49	0.873	(0.738–1.032)	0.1108	0.897	(0.757–1.062)	0.2068
50–69 (reference)	1			1		
≧70	2.025	(1.873–2.189)	<0.0001	1.424	(1.308–1.550)	<0.0001
Gender						
Male (reference)	1	–		1		
Female	0.577	(0.530–0.628)	<0.0001	0.739	(0.673–0.812)	<0.0001
CCI score						
≦2 (reference)	1	–		1		
3–5	1.312	(1.213–1.419)	<0.0001	1.118	(1.033–1.211)	0.006
>5	1.553	(1.305–1.850)	<0.0001	1.251	(1.047–1.494)	0.0137
Cell type						
AD (reference)	1	–		1		
SqCC	1.946	(1.801–2.103)	<0.0001	1.327	(1.213–1.452)	<0.0001
LCC	1.963	(1.665–2.314)	<0.0001	1.569	(1.313–1.874)	<0.0001
Others	1.153	(0.928–1.433)	0.1976	1.335	(1.071–1.665)	0.0102
Grade						
Well differentiated (reference)	1			1		
Moderately differentiated	1.971	(1.586–2.450)	<0.0001	1.590	(1.275–1.983)	<0.0001
Poorly differentiated	2.527	(2.030–3.147)	<0.0001	1.869	(1.492–2.341)	<0.0001
Undifferentiated	2.314	(1.440–3.720)	0.0005	1.691	(1.028–2.782)	0.0386
Unknown	3.421	(2.764–4.235)	<0.0001	1.797	(1.441–2.242)	<0.0001
TN stage						
T1N2 (reference)	1			1		
T2N2	1.570	(1.363–1.808)	<0.0001	1.209	(1.047–1.396)	0.0098
T3N1	1.620	(1.370–1.917)	<0.0001	1.171	(0.985–1.392)	0.0737
T3N2	2.211	(1.914–2.553)	<0.0001	1.411	(1.214–1.639)	<0.0001
T4N0	1.285	(1.095–1.507)	0.0021	0.998	(0.849–1.174)	0.9838
T4N1	2.075	(1.730–2.489)	<0.0001	1.270	(1.053–1.533)	0.0125
Treatment						
None (reference)	1			1		
OP alone	0.182	(0.153–0.216)	<0.0001	0.349	(0.266–0.458)	<0.0001
Neoadjuvant + OP + adjuvant	0.181	(0.151–0.218)	<0.0001	0.322	(0.243–0.427)	<0.0001
OP + adjuvant	0.211	(0.185–0.241)	<0.0001	0.397	(0.311–0.506)	<0.0001
CRT	0.511	(0.438–0.597)	<0.0001	0.486	(0.415–0.570)	<0.0001
CT ± target therapy	0.728	(0.639–0.830)	<0.0001	0.741	(0.649–0.846)	<0.0001
RT alone	0.910	(0.776–1.067)	0.2469	0.801	(0.682–0.941)	0.007
Target therapy	0.436	(0.366–0.521)	<0.0001	0.649	(0.540–0.781)	<0.0001
Others	0.738	(0.601–0.905)	0.0035	0.707	(0.576–0.868)	0.0009
OP method						
None (reference)	1			1		
Wedge resection	0.343	(0.289–0.408)	<0.0001	0.866	(0.664–1.131)	0.2907
Segmentectomy	0.139	(0.077–0.252)	<0.0001	0.402	(0.214–0.755)	0.0046
Lobectomy	0.279	(0.256–0.304)	<0.0001	0.634	(0.508–0.790)	<0.0001
Bilobectomy	0.329	(0.244–0.444)	<0.0001	0.681	(0.473–0.981)	0.0390
Pneumonectomy	0.409	(0.301–0.556)	<0.0001	0.833	(0.572–1.212)	0.3395

Abbrevations: AD, adenocarcinoma; CI, confidence interval; CCI, charlson comorbidity index; CRT, chemoradiotherapy; CT, chemotherapy; HR, hazard ratio; LCC, large cell carcinoma; OP, operation; RT, radiotherapy; SqCC, squamous cell carcinoma.

## 4 DISCUSSION

In this study, we demonstrated that the different heterogenicity of stage IIIa NSCLC contributed to different outcomes. Factors that predicted better OS in stage IIIa NSCLC patients were young age, female gender, lower CCI score, AD, well differentiated, and T1N2/T4N0. We also noted that patients received surgical treatment revealed much better outcomes than those did not.

There were some studies that compared the role of operation in the treatment of stage IIIa NSCLC. The EORTC 08941 trial pointed out that surgical resection did not improve OS or progression‐free survival (PFS) compared with radiotherapy after induction chemotherapy in stage IIIA NSCLC.^12^ In the Intergroup 0139 trial, concurrent chemoradiotherapy (CCRT) following surgery yielded better PFS than definitive CCRT in T1‐3N2M0 NSCLC patients (12.8 vs. 10.5 months). The OS was better for patients who received lobectomy, but not for those who received pneumonectomy, compared to definitive CCRT.^7^ The ESPATUE trial indicated that chemoradiotherapy, either with or without surgery, provided acceptable outcomes in the treatment of stage IIIa and IIIb NSCLC.^13^ In this study, stage IIIa NSCLC patients who received surgery had significantly better OS than those who did not. This may result from several factors. First, over 70% of the operations in Taiwan were lobectomies/segmentectomies. In conformity with the Intergroup 0139 trial, our study also showed better OS with lobectomy than with pneumonectomy or wedge resection. With improvements in surgical techniques and the higher proportion of lobectomies and segmentectomies, the surgical outcomes might have improved as time went on. Second, the abovementioned trials only enrolled patients with N2 disease while our study consisted of all stage IIIa patients. Stage IIIa patients with T4N0, T4N1 and T3N1 might have a better surgical response since they had a more locally advanced disease. The tumor was confined within the pleural cavity and did not spread to the mediastinum.

While the standard surgical treatment of stage IIIa NSCLC remained lobectomy, pneumonectomy and sublobar resection were examined in several studies. Concerning pneumonectomy, high 30‐day mortality rates ranging from 4.2% to 13.3% were reported.^14^, ^15^, ^16^ Some studies suggested that it should be only considered in selected patients.^7^, ^14^ On the other hand, other studies indicated that pneumonectomy provided acceptable long‐term survival and should not be excluded for selected patients with stage IIIA‐N2 NSCLC.^15^, ^16^, ^17^ There are fewer studies of sublobar resection for stage IIIa NSCLC. Only one cohort study with 21,638 stage IIIa/N2 NSCLC patients from the Surveillance, Epidemiology, and End Results (SEER) database compared no surgery, sublobar resection, and lobectomy. Lobectomy provided significantly better OS than sublobar resection for patients less than 65 years old, but no difference was noted in patients over 65 years old.^18^

It is interesting that segmentectomy was observed to result in significantly better OS than other surgical strategies in this study. No study compared the outcomes of lobectomy and segmentectomy in stage IIIa NSCLC patients. The indications of segmentectomy in Taiwan might include poor pulmonary function or peripheral nodule less than 2 cm and no strong evidence of positive N2 lymph node stations. In this study, only 56 out of 2005 (2.8%) resections were done by segmentectomy. These patients were highly selected and not quite common in stage IIIa NSCLC. In our study, the reason why segmentectomy provided better OS than lobectomy might be explained as follows. There might be some bias in that most of the patients who received segmentectomy were stage T1N2, which had the best prognosis in all stage IIIa NSCLC patients. Lobectomy was performed in the T and N stages with worse outcomes. Further studies focusing on the T1N2 stage comparing segmentectomy and lobectomy may need to be conducted. We suggest that segmentectomy might be performed in selected patients with T1N2 NSCLC and result in non‐inferior outcomes compared to lobectomy.

The outcomes of heterogeneity in stage IIIA NSCLC were discussed in recent research. A study enrolled patients with pathological stage IIIa NSCLC who received operations in Istanbul and Zurich.^19^ There were 308 patients assigned to the stage IIIA‐T group (T4N0–1 & T3N1), and 116 patients were assigned to the stage IIIA‐N2 group (T1‐2N2). The OS was much better in the stage IIIA‐T group than in the stage IIIA‐N2 group. Our study held a differing view that T1N2 heterogeneity had the best outcomes in stage IIIA NSCLC patients, followed by T4N0 and T3N1/T2N2. These different outcomes might be caused by differences in patient inclusion criteria and therapeutic strategies. The stage IIIA NSCLC patients had regional differences and received a variety of treatments.

For stage IIIa NSCLC, trimodality and bimodality treatments involving surgery, chemotherapy and radiotherapy were well discussed. Previous studies more than two decades ago laid the foundation of induction treatment followed by surgery rather than surgery alone.^20^, ^21^, ^22^ Several large trials, including GLCCG, WJTOG9903 and SAAK, further concluded that there is no benefit for additional radiotherapy as induction treatment for stage IIIa‐N2 NSCLC.^8^, ^10^, ^23^ In recent years, the outcomes of trimodality treatment and bimodality treatment have been discussed from several points of view.^6^, ^24^ It seems that trimodality treatment might provide slightly better outcomes than bimodality treatment in selected patients with stage IIIa NSCLC. However, the neoadjuvant therapy is not quite common for clinical N2 NSLCL patients in Taiwan. Most patients received direct operation with adjuvant therapy if the tumor is resectable. The rate of direct operation with adjuvant therapy is 39.3% in cT1N2, 31.5% in cT2N2, and 17.1% in cT3N2, respectively. Most of the patients received definite therapy when the tumor is considered unresectable. The neoadjuvant therapy rate in this study is only 9.4% in cT1N2, 11.2% in cT2N2, and 11.1% in cT3N2, respectively.

In this study, we also noticed that stage IIIa NSCLC patients who followed guideline treatment showed better outcomes than those did not. However, most patients who did not follow the guideline treatment might due to poor performance status, poor pulmonary function or combination of several comorbidities. It is still hard to conclude the overall effect of guideline treatment.

A strength of our study is that the inclusion of a large number of patients provided accurate statistical analyses and detailed analysis of the heterogenicity in stage IIIa NSCLC patients. In addition, we noticed that it is interesting to focus on the treatment of T1N2 patients and the role of segmentectomy in the future. However, there were several limitations in this study. First, the retrospective design may have inserted a selection bias, which could affect the outcomes. The confounders are difficult to avoid and prospective randomized control trial is needed to make concrete conclusions. Second, the study period was between 2010 and 2018, so some of the data was based on the 7th American Joint Committee on Cancer (AJCC) TNM staging system and other data was based on the 8th AJCC TNM staging system. Further studies with data focused on the 8th AJCC TNM staging system are needed to confirm the results. Third, the evaluation of lymph node station data is not very detailed. Asamura et al. classified the N2 lymph node into N2a1 (single skip metastasis), N2a2 (single without skip metastasis) and N2b (multiple N2 station), and the outcomes were different.^25^ The different N2 lymph node metastases also need to be discussed as well. Fourth, we do not get the PET/CT and Endobronchial Ultrasound data in this study. The correlation between clinical and pathological stage is uncertain. The clinical stage of stage IIIa NSCLC might not be highly accuracy in Taiwan. Last but not least, the TSCR does not include immunotherapy data. As a result of the growing trend of combining immunotherapy into the multiple modality treatments, the outcomes have improved rapidly. The PACIFIC trial and SAKK 16/14 trial focused on the effect of durvalumab in stage III NSCLC patients.^26^, ^27^ It is important to consider these therapies in future analysis.

In conclusion, the heterogenicity of T1N2 resulted the best outcome of clinical stage IIIa NSCLC. Patients received surgical treatment revealed much better outcomes than those did not. Lobectomy is the standard choice of surgical treatment and multimodal therapies with individualized treatment tend to provide better survival outcomes.

## AUTHOR CONTRIBUTIONS

**Ya‐Fu Cheng:** Writing – original draft (lead). **Jing‐Yang Huang:** Methodology (lead); software (lead); validation (lead). **Ching‐Hsiung Lin:** Data curation (lead); investigation (lead); visualization (lead). **Bing Yen Wang:** Conceptualization (lead); writing – review and editing (lead).

## FUNDING INFORMATION

The authors received no specific funding for this work.

## CONFLICT OF INTEREST STATEMENT

The authors declare no conflict of interest.

## ETHICS STATEMENT

The study was approved by the Institutional Review Board in our institution (Changhua Christian hospital, IRB‐221211), and informed consent from all participants was waived.

## Data Availability

The datasets generated and/or analyzed during the current study are available from the corresponding author on reasonable request.
